# IRRISENS: An IoT Platform Based on Microservices Applied in Commercial-Scale Crops Working in a Multi-Cloud Environment

**DOI:** 10.3390/s20247163

**Published:** 2020-12-14

**Authors:** Rodrigo Filev Maia, Carlos Ballester Lurbe, Arbind Agrahari Baniya, John Hornbuckle

**Affiliations:** Centre of Regional and Rural Future, Deakin University, Hanwood 2680, Australia; carlos.ballesterlurbe@deakin.edu.au (C.B.L.); a.agraharibaniya@deakin.edu.au (A.A.B.); j.hornbuckle@deakin.edu.au (J.H.)

**Keywords:** IoT platform, microservice, smart agriculture, irrigated crops, agriculture 4.0

## Abstract

Research has shown the multitude of applications that Internet of Things (IoT), cloud computing, and forecast technologies present in every sector. In agriculture, one application is the monitoring of factors that influence crop development to assist in making crop management decisions. Research on the application of such technologies in agriculture has been mainly conducted at small experimental sites or under controlled conditions. This research has provided relevant insights and guidelines for the use of different types of sensors, application of a multitude of algorithms to forecast relevant parameters as well as architectural approaches of IoT platforms. However, research on the implementation of IoT platforms at the commercial scale is needed to identify platform requirements to properly function under such conditions. This article evaluates an IoT platform (IRRISENS) based on fully replicable microservices used to sense soil, crop, and atmosphere parameters, interact with third-party cloud services for scheduling irrigation and, potentially, control irrigation automatically. The proposed IoT platform was evaluated during one growing season at four commercial-scale farms on two broadacre irrigated crops with very different water management requirements (rice and cotton). Five main requirements for IoT platforms to be used in agriculture at commercial scale were identified from implementing IRRISENS as an irrigation support tool for rice and cotton production: scalability, flexibility, heterogeneity, robustness to failure, and security. The platform addressed all these requirements. The results showed that the microservice-based approach used is robust against both intermittent and critical failures in the field that could occur in any of the monitored sites. Further, processing or storage overload caused by datalogger malfunctioning or other reasons at one farm did not affect the platform’s performance. The platform was able to deal with different types of data heterogeneity. Since there are no shared microservices among farms, the IoT platform proposed here also provides data isolation, maintaining data confidentiality for each user, which is relevant in a commercial farm scenario.

## 1. Introduction

Irrigated agriculture is a key industry to meet the food requirements of a constantly increasing world population in which any productivity improvement has direct benefits on society and the environment. In the last decades, the application of computational technologies in agriculture has increased dramatically, resulting in increased productivity and more efficient use of natural resources. Optimization of irrigation management is crucial in semiarid areas where water scarcity is a serious issue. The application of computational technologies in these areas may not only have a beneficial impact on farm management and cost efficiencies, but also on the availability of water resources for other crops or even sectors [1]. As agriculture demands 70% of freshwater in current food production systems [2], saving and optimizing water resources in this sector is imperative to achieving the Food and Agriculture Organization (FAO) expectation to double food production by 2050 [3].

The application of information and communication technologies in agriculture is represented by several concepts, such as precision agriculture (PA) [4] and smart farming [5]. However, as discussed in [6], such concepts have a broad interpretation which makes the use of these terms confusing. In an industrial agriculture scenario, the application of technology considers not only data acquisition and monitoring but also an integration of services and impacts in the business process that it is close to the concept of Industry 4.0 [7]. That is, the development of a system has a maturity chain comprising six levels: computerization, connectivity, visibility, transparency, predictive capability, and adaptability [8]. In the work presented here, the application of Internet of Things (IoT), smart sensing [9], big data, and services, and its integration is discussed in order to provide elements to farm automation and decision-making following the paradigm of Agriculture 4.0 [10]. In the context of Agriculture 4.0, several IoT platforms have been proposed to monitor, control, and forecast crop parameters. Monitoring and control will entail the first three levels of the maturity chain for the development of a system (computerization, connectivity, and visibility). Forecasting parameters, on the other hand, would represent the first stages of the fourth level, transparency, considering that forecasting helps to understand the crop scenario and cannot be used to simulate crop scenarios as suggested in the fifth level of the maturity model [8].

Several system architectures (platforms) have been proposed to deal with some of the challenges presented in agriculture. Some platforms are cloud-centric, where the smart sensing devices installed in the field send data to a remote central processing system that may even support decision making [11,12,13,14]. Other IoT platforms rely on hybrid structures composed of edge or fog computing and cloud computing useful to deal with communication shortcomings between devices and the cloud [15,16,17]. Table 1 classifies several of the IoT platforms applied in agriculture, classified based on two major categories: organization (how the platform is organized in a computation environment) and software architecture, which determines the flexibility and adaptability of a platform. The classification took into consideration the fog computing definition “as an extension of cloud computing to the edge of the network” [18]. It implies that the fog has the same basic structure of the cloud but exist in the farm to deal with communication latency among other issues [19,20]. Edge computing is understood as “as any computing and network resources along the path between data sources and cloud data centres” [21]. While interesting for specific purposes, platforms composed of edge or fog computing are challenging to interact with external services such as remote-sensed data providers or weather forecast services that provide relevant information, such as the normalized difference vegetation index (NDVI) or the reference evapotranspiration (ETo) for irrigation scheduling decisions. Indeed, in most platforms, the interactions with external services are performed by the cloud.

IoT platforms may be based on software frameworks such as FIWARE [14,16,22], proprietary or custom-made software frameworks following a multitude of software architectures [23] or microservices-based architecture [24,25,26,27]. FIWARE makes interoperability between applications called enablers and has the context broker ORION in its core. The context broker is the element to orchestrate communication between all enablers and external services using the NGSI protocol [28] or an IoT agent in case NGSI is not present. One of the concerns about FIWARE is the difficulty to isolate data from different sources while using the ORION context broker, since it uses only one database to store data from all services and this may compromise data isolation. In [29], the usage of one context broker for each farm has been discussed in the case of a commercial-scale scenario. This, however, implies that it would have several instances of the platform instead of one. Implementation of these IoT platforms has been evaluated in experimental sites or under controlled conditions (greenhouses) [30,31]. Although these studies provide relevant insights and guidelines about architectural approaches for IoT platforms, proper use of sensors, and application of algorithms for forecasting relevant parameters, more research is needed to evaluate IoT platforms that could deal with the actual requirements of farms at a commercial scale. When multiple farms are monitored at the same time, but they must be managed independently, a microservice-based architecture could present advantages compared to a monolithic architecture. However, only a few studies have reported IoT platforms designed with a microservice-based architecture [23,24,32].

**Table 1:** Internet of Things (IoT) platforms in an agriculture context.

Organization			Software Architecture		References
Cloud	Fog	Edge	Monolithic	Microservices	
Application- specific and generic services			Application- specific	Generic services	[11,14,22]
Application- specific and generic services	Application- specific		Application- specific	Generic services	[16]
Application- specific and generic services	Application- specific		Generic services	Application- specific	[15,24]
Application- specific and generic services			Generic services	Application- specific	[32]
All software runs here			Platform and application- specific		[33,34,35,36,37,38,39,40]
Data processing and specific services		Application- specific	Application- specific		[13]
Application- specific services	Application- specific services	Application-specific			[17]
Application- specific and generic services			Generic services	Application- specific	[23]

**Figure 1:**
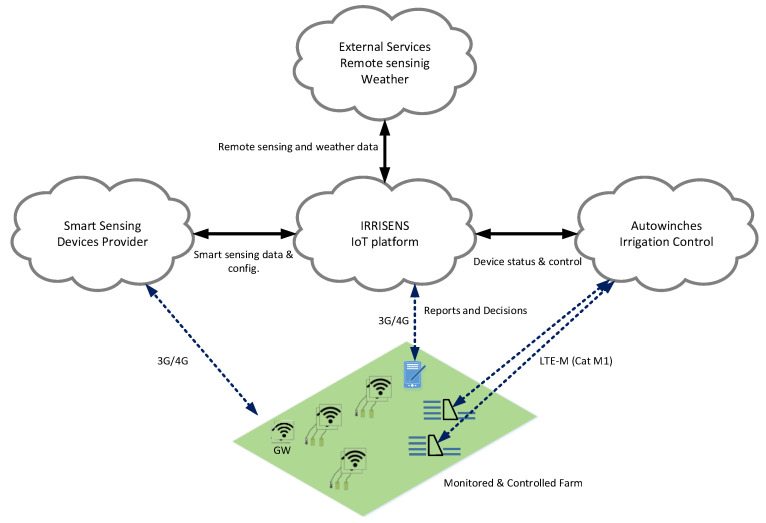
Schematic diagram representing the interaction between the IoT platform presented in this study (IRRISENS) and the external services.

The overall objective of this work was to evaluate the requirements and feasibility of using an IoT platform based on fully replicable microservices for collecting soil, crop, and weather data from different sources as well as interacting with external (weather and remote-sensed data providers) and third-party cloud services for irrigation scheduling decision support and, potentially, irrigation automation at a commercial scale. The IoT platform IRRISENS shown in Figure 1, which was developed in this research, was evaluated on broadacre irrigated crops (rice and cotton) by monitoring at four commercial farms. The robustness of the platform was evaluated and the requirements of an IoT platform for irrigation scheduling decision support were identified.

This paper is organized as follows: Section 2 presents the materials and methods, in which the scenario where the platform was tested and how the performance and robustness of IRRISENS was evaluated are described. Section 3 describes the IRRISENS platform architecture. In Section 4, the results obtained from validating and evaluating the platform at commercial-scale farms are presented. Section 5 and Section 6, respectively, discuss the performance of the platform and provide the conclusions and future research needs proposed based on this study.

## 2. Materials and Methods

### 2.1. Commerical Scale Field Trails of IRRISENS

**Figure 2:**
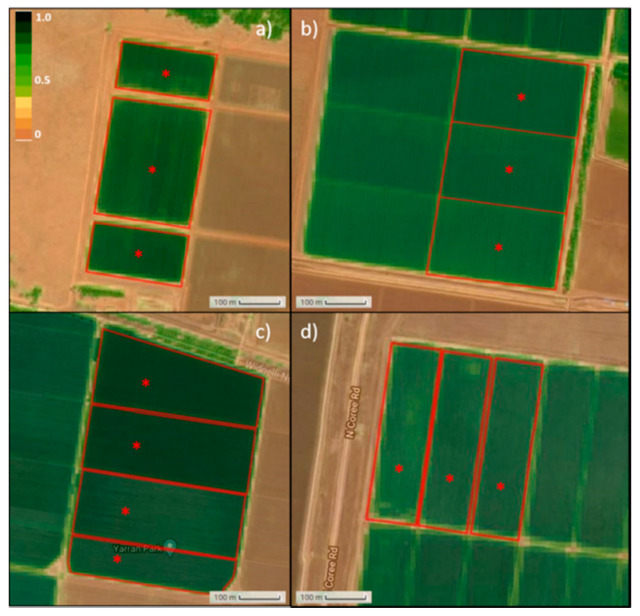
Sentinel-2 normalized difference vegetation index (NDVI) image (obtained from IrriSat [42]) of the cotton (**a**,**b**) and rice farms (**c**,**d**) with detail of the bays monitored (red polygons) and location of the sensors (red asterisks) at each site.

IRRISENS was evaluated in the 2019/20 summer season in two commercial cotton and two rice farms located in the Murrumbidgee Valley in NSW, Australia. A Wi-Fi network consisting of a cellular data modem connected by ethernet to an Ubiquiti Nanostation M2 directional Wi-Fi access point was installed at each site to provide internet connectivity to the monitored bays (3–4 bays per site; see Figure 2). IoT Wi-Fi-based loggers (WiField, Goanna telemetry, Goondiwindi, QLD, Australia) were used at all the sites to collect and send data from the sensors to the platform every hour [41]. Each bay was equipped with irrigation outlets and automation units (Autowinch Seasonal, Padman Automation, Strathmerton, Victoria, Australia) to control water distribution. Both WiField loggers and irrigation control devices used a cloud service with different field communication technology with its protocols, data organization, and communication APIs (Application Programming Interface) to collect data and interact with field equipment. The platform was evaluated by collecting and processing data from the different sources (in-field sensors, weather stations and remote-sensing provider platform) and making the processed data available for farmers/agronomists in a user-friendly interface for them to make decisions regarding when to irrigate.

#### 2.1.1. Cotton

**Figure 3:**
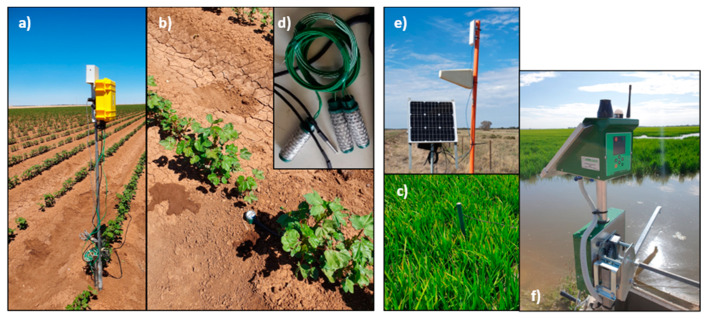
Pictures of a datalogger (**a**), multisensor capacitance probes installed in a cotton and rice farm (**b**,**c**), Watermark sensors (**d**), a Wi-Fi access point (**e**), and an IoT irrigation control autowinch (**f**).

Cotton was furrow irrigated by means of a bank-less channel irrigation system. Soil moisture was monitored at the centre of three contiguous bays with a multisensor capacitance probe (EP100G-12, Entelechy Pty Ltd., Golden Grove, SA, Australia) with sensors at 0.10 m intervals along the length of the probe (1.2 m) that collected volumetric water content, temperature, and salinity data [43]. Soil water tension (kPa) was also monitored at 0.20 m below the surface using a pair of Watermark sensors (model 6440 Davis Instruments, United States) and a temperature sensor (DS18B20, Maxim Integrated) using a standard equation [44] (see the detail of the sensors in Figure 3). Compared to the capacitance probes, the Watermark sensors do not need calibration for different soils and provide a low cost and a simple way for determining when soil is reaching limiting moisture levels that could affect plant physiology. Table 2 summarizes the parameters monitored at the cotton and rice farms.

#### 2.1.2. Rice

Rice was grown following a delayed permanent water strategy where the crop is not permanently flooded until late tillering. Like in the cotton farms, multisensor capacitance probes were installed in 3–4 contiguous bays. In this case, however, soil moisture was not a relevant parameter to monitor because sensors were installed in January when bays had been already flooded. The capacitance probes were used instead for measuring water height in the ponded bays according to [45]. Probes were 0.8 m long and were installed to leave the first 0.6 m from the top above the soil surface to be able to monitor water height but also soil, water, and air temperature at the canopy level. This case in which the same type of sensor is used to measure different parameters (volumetric water content and water height) and only one of them (water height in this case) is relevant for the irrigation management at a specific crop stage is an example of data heterogeneity.

### 2.2. Identifying IoT Platform Requirements at Comerical Scales

In order to identify the IoT platform requirements at the commerical scale, end users (irrigators and farmers) were consulted before, during, and after the experimental period to capture important elements and requirements such an IoT platform would need to meet expectations and requirements of these industries. This was undertaken through structured meetings with these end users. Experience gained by the users and researchers during this implementation period was used to develop a set of key requirements that are recommended for IoT platforms when aiming to be used in agriculture at commercial scales when a multitude of farms are to be monitored simultaneously, based on this experience and feedback.

### 2.3. Evaluating Platform Robustness

Evaluation of the robustness of the platform was performed by comparing for 46 days the impact that a failure occurring at one of the farms, in which a datalogger collected and sent data every 20 s instead of 1 h, could have on the execution response time (latency) of the “crop parameter” ASS when configured to run as a microservice, as it is configured in IRRISENS, and when configured as a monolithic service that processes data for all the monitored farms. Since IRRISENS is running on a commercial cloud, it was not relevant to evaluate the latency of the General Cloud Services, which would be related to the cloud infrastructure rather than to the platform itself. Instead, the variability in daily latency of a microservice was monitored during a month and the effect of data load on latency was assessed.

## 3. Description of the IRRISENS IoT Platform

**Figure 4:**
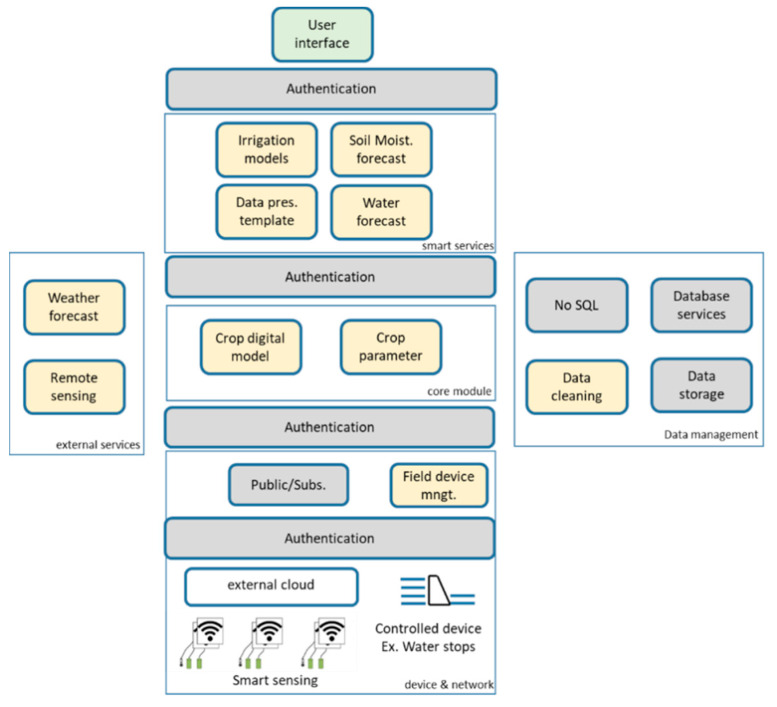
Modules and microservices that compose the IRRISENS IoT platform. Application-specific services (ASS) directly related to agriculture are indicated in yellow colour while general cloud services (GCS) are indicated in grey colour.

IRRISENS is a cloud-centric platform composed of two different types of software elements: native cloud services and microservices. A microservice is by definition an independent process that performs a specific task and communicates by employing a lightweight mechanism with minimum centralized management [46,47]. Further, it can be “deployed, changed, substituted, and scaled independently of each other” [48], as opposed to monolith software where “modules cannot be executed independently” [46]. These elements (native cloud services and microservices) provide domain-specific services all related to the agriculture context and are organized in six modules as depicted in Figure 4. Each module is composed of fully replicable services and microservices that deal with sensing data, monitoring farm status, controlling irrigation, and forecasting decision-making features, data management, and presentation, as well as integration with external services. The platform interacts with external services running on their clouds to monitor and control IoT devices already installed in the farms, which operate with their messaging based on both open standards and proprietary communication protocols. Some services are based on a publish/subscribe mechanism and others are based on external APIs using MQTT, JSON, or REST.

The services not specifically related to the agriculture domain but essential to the IRRISENS platform, are called General Cloud Services (GCS), and in the cloud, there are controls about their performance and configuration. The GCS present native cloud features such as elasticity, which means the cloud will provide enough resources to execute the service and process data regardless of the data load imposed by the service. The services related directly to agriculture are called Application Specific Services (ASS) and they run according to the resources and features configured by the IRRISENS administration user, which directly affects the IoT platform’s performance. The ASS are microservices specialized for agriculture tasks and, thus, must be robust and capable to deal with data heterogeneity, data processing, forecast and device control. In this work, all ASS ran with the same resource configuration and without elasticity, i.e., each ASS will run with the same amount of configured resources from the cloud platform. All GCS and ASS were configured to run with authentication between them and other services and microservices. This guarantees data isolation between farms.

### 3.1. Platform Organization

The IRRISENS core module is composed of the “crop digital model” and “crop parameter” microservices (Figure 4). All the microservices in the platform refer to the “crop digital model” microservice to perform its processing. The “crop digital model” microservice is based on the evapotranspiration model [49,50] and it is specific for each crop. The “crop parameter” microservice is responsible for collecting data from the available sources (soil moisture sensors, weather stations, and remote sensing services) to fulfil the model. It also must deal with data heterogeneity. The “crop digital model” is a fundamental element in the platform since it describes the physical crop under monitoring. The model not only provides the parameters to be collected by the sensors, but as it is related to a specific type of crop, it also determines what data means and how to interpret the sensors’ readings, and, thus, gives support to deal with data heterogeneity.

The IoT device and network module is composed of services and microservices to interact with smart sensing devices, control devices, and external clouds that control other devices in the field that IRRISENS must interact with. In this module, the use of both GCS and ASS is essential to deal with several protocol communication technologies and provide platform scalability and robustness. IRRISENS may use the publish/subscribe services and microservices dedicated to deal with smart sensing devices. The “field-device management” microservice deals with labelling and format of data collected from the field before data is processed by the ASS “crop parameter” and stored in the GCS “data storage” microservice. Once the data collected are formatted according to the platform requirements, these will be processed by the “crop parameter” microservice.

In the external services module, the ASS microservices are responsible for the exchange and collection of data from external data providers, such as remote sensing platforms and weather services. There is a specific microservice for weather forecast and other for remote sensing. Both microservices are instantiated (i.e., start running the microservice) for each farm following specifications about which data must be collected at each farm. Examples of data specification are GPS coordinates of a farm to receive weather forecast from the nearest weather station available and crop reflectance data obtained from satellites used to compute the NDVI. These microservices (“weather forecast” and “remote sensing”) deal with data at different spatial and temporal resolutions and organize data according to the storage requirements of IRRISENS. An example of this spatial and temporal data heterogeneity is data from weather stations collected from a specific farm at hourly intervals, data from weather forecast services for large areas collected daily, and data from satellite imagery collected at five-day intervals.

The module Data Management has a “database services” and “data storage” microservices that manage data persistency in the platform. All microservices from this module perform the basic data processing such as cleaning data and storing data in raw format. In some cases, sensors do not provide a ready-to-use value and raw data collected need to be processed to obtain a meaningful value with a specific unit. That is, for instance, the case of the Watermark sensors. The sensor measures the resistance value (*ohms*) that needs to be converted to soil water tension (kPa), a measure of the energy required to extract water from the soil. In this case, the platform stores the raw data to evaluate whether there are possible anomalies in the measurement and then, the “digital crop model” ASS calculates the soil water tension according to the sensor manufacturer’s equation.

The Smart Services module is composed of four microservices that run simple or complex algorithms (machine learning algorithms) to process data from the lower modules and compose them to provide services to farmers. That is, for instance, soil moisture forecast and calculation of crop parameters or models to estimate crop water needs, which are useful for the irrigation scheduling decision-making process and, potentially for controlling field equipment such as the irrigation control winches responsible to control water distribution during an irrigation event.

The Integrated Service is the module where the interface between final users and the IoT platform is managed. The elements in this module control user authentication, data visualization for monitoring, and interfaces to control actuators in the field.

The ASS may be organized and instantiated for each monitored crop and GCS can be securely shared between ASS to make it possible to have a simultaneous set of microservices instances in the same structural element (the cloud) to provide customized behaviors according to instantiation (code into computer memory ready to be executed) of selected microservices. As all microservices must be authenticated by a cloud platform, there is an important level of security that guarantees one microservice instantiated for a farm does not interact with microservices or data from other farms. The microservice-based architecture also provides a better computational resource allocation, since each microservice is an independent service to which the cloud allocates an amount of memory space and processing time.

The crop digital model runs periodically to process data and update the crop model. It requests authentication to access repositories to collect and process all data regarding the crop. As several readings come from sensors that asynchronously send data to the platform, the ASS running periodically can take all data sent in an interval of time and consolidate all values to represent the crop status.

### 3.2. Data Isolation Approach and Data Flow

Commercial farms require robust IoT platforms and data isolation as the collected data in the platform is sensitive for the farm business. Crop and irrigation management are directly related to crop yield. Therefore, the platform must provide security mechanisms that avoid possible breaches that could compromise data secrecy.

**Figure 5:**
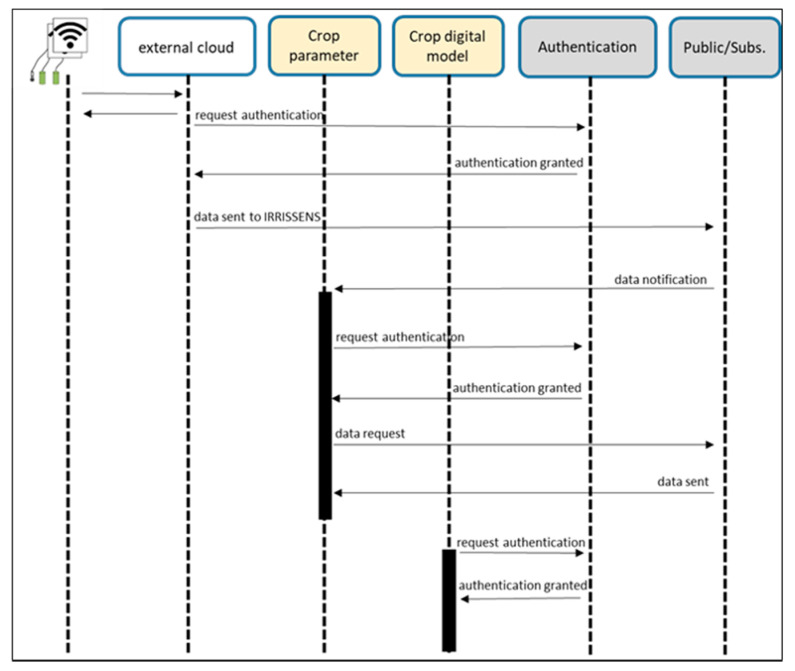
Mechanisms of authentication among all elements of IRRISENS.

In IRRISENS, security is based on authentication and security certificate among all elements of the platform, regardless of internal or external services. As illustrated in Figure 5, every ASS has the credentials to run and access any GCS. Those credentials are created and configured by the platform administrator. These may be associated with all ASS running for one farm, which creates a chain of authenticated ASS that manipulates data.

**Figure 6:**
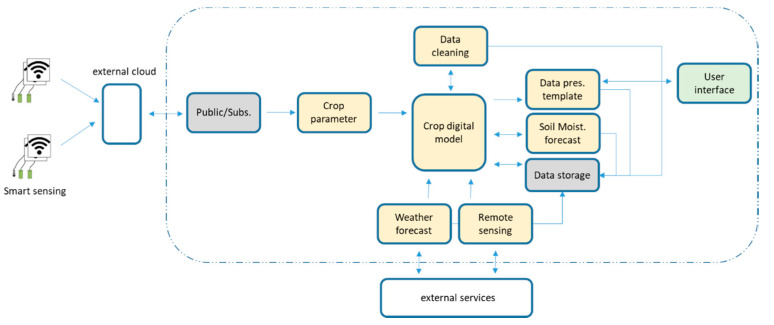
Dataflow diagram with an example of the organization of the General Cloud Services (GCS; grey colour) and Application Specific Services (ASS; yellow colour) within the platform for the monitoring of a cotton or rice farm.

Figure 6 illustrates a dataflow diagram with an example of the organization of the GCS and ASS services valid for the monitoring of a cotton and rice farm. The microservice “crop parameter” collects all data coming from the sensors in the field and organize these in a way that can be read by the “crop digital model” microservice. Once the farm and type of crop are identified by the “crop digital model” microservice, all the data is subjected to a data cleaning process (“data cleaning” microservice) where abnormal readings from the sensors are detected and data is made available for the “crop digital model” microservice to process accordingly. The “crop digital model” microservice uses then data from the closest available weather station, including air temperature and humidity, wind speed, solar radiation and precipitation, to calculate the reference evapotranspiration (ET_o_) according to [49] and remotely sensed NDVI obtained from Sentinel-2 imagery to determine a site-specific crop coefficient (K_c_) according to [51] and estimate the crop evapotranspiration (ET_c_ = K_c_ × ET_o_). The “soil moisture forecast” microservice in cotton and “volume water forecast” microservice in rice (see Figure 6), use this digital representation to forecast soil water tension and volume of water, respectively, at each bay for the next seven days to support irrigation scheduling decisions. Finally, the “data presentation template” microservice organizes data in a user-friendly way for the users to consult, evaluate, and make the pertinent decisions. Currently, the platform does not provide any type of alert or notification to the users when a problem occurring in the field prevents data to be collected and processed by the platform. Nevertheless, the users of the platform can visually detect when this situation occurs in the user interface where the most relevant parameters are presented.

### 3.3. Data Heterogeneity

In the context of the IRRISENS platform, data heterogeneity refers to three aspects: (i) the interface between smart sensing devices and IRRISENS, and between the platform and external services (i.e., different interfaces and protocols between the external services); (ii) the use of data collected from the same type of sensor to monitor different parameters according to the crop being monitored, and; (iii) the spatial and temporal resolution of data to be used to compose the IoT platform results. An example of data heterogeneity related to sensor data in the context in which IRRISENS was evaluated is the use of the multisensor capacitance probes. While in cotton farms these probes were used to monitor volumetric water content, in rice farms where the crop was ponded and soil remained saturated, raw data from the sensors were used to calculate the water height within each bay. Having data from sensors deployed in the field available every hour, from weather stations and data from Google Earth Engine as in the case of the Sentinel-2 NDVI satellite imagery available every five days for computing crop evapotranspiration is another example of heterogeneity in temporal resolution.

The heterogeneity between IRRISENS and devices is handled by the “field device management” ASS and its function is similar to that of a FIWARE IoT-Agent [28,29] as presented in [16]. The IRRISENS platform relies on the “crop parameter” ASS to deal with data format heterogeneity to exchange data with external services through a public subscriber mechanism or proprietary API. After the “crop parameter” microservice processes all data from the different sources and organizes data with the same format, the “crop digital model” computes data according to the crop type to deal with heterogeneities (ii) and (iii).

The use of ontologies can assist the platform in dealing with heterogeneity [52]. However, the ontology was not considered at this stage of the IRRISENS conception because there is no clear definition about what should be a syntactic and semantic structure for data in the commercial-scale farms and none of the external services considered in this work follows any defined ontology.

## 4. Results

**Figure 7:**
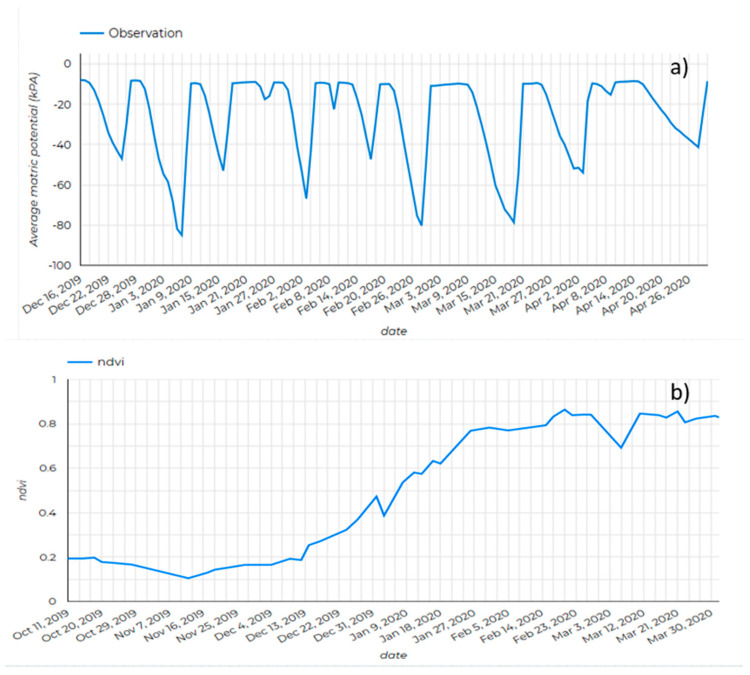
Soil matric potential (**a**) and the normalized difference vegetation index (NDVI) (**b**) monitored in one of the cotton farms over the crop season.

**Figure 8:**
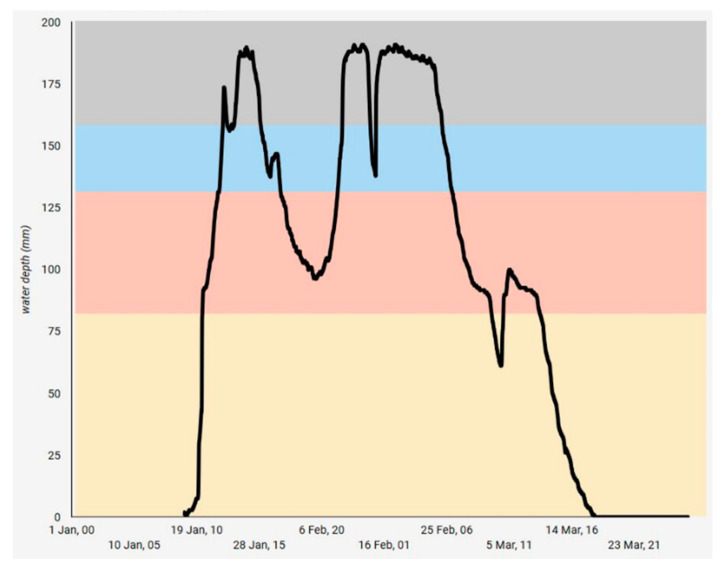
Daily water height monitored in a bay of a rice farm obtained from the multisensor capacitance probes.

As part of the evaluation of the feasibility of using IRRISENS for irrigation scheduling decision support, the performance of the platform regarding monitoring soil, crop, and atmosphere parameters is presented here. Once sensors were deployed in the field, IRRISENS started collecting data from the set of sensors installed at each farm as well as from external services at the specified time intervals. This information was useful for the farmers/agronomists to make decisions regarding the water management of each crop. Figure 7 and Figure 8 illustrate the type of data generated by the “crop digital model” microservice and how these data are made available to the farmer/agronomist.

In cotton, continuous monitoring of soil water tension and the seven-day soil water tension forecast enabled the farm/agronomists responsible for the farms to schedule irrigation avoiding values below −60 kPa, when cotton plants start showing symptoms of water stress [53] (Figure 7a). As expected, the remotely sensed NDVI obtained from an external service presented low values early in the growing season, when most of the pixels in the satellite image contained bare soil, and increased progressively up to ~0.90 when crops reached full ground canopy cover (Figure 7b). Likewise, water requirements (ETc) of crops increased along the growing season as biomass increased.

In rice, despite the capacitance probes being the same type as those used in the cotton farms, the “crop digital model” microservice computed water height instead of volumetric water content, showing the capacity of the platform to deal with data heterogeneity across sensors (Figure 8). Monitoring water height was relevant for the water management in rice farms for two reasons. Rice is sensitive to low temperatures at the microspore stage when temperatures below 17 °C can damage the pollen and lead to floret sterility [54]. Since temperature changes in water take longer to occur than in the air, water depths of 0.25 m have been recommended at the microspore stage to protect the crop against low temperatures [55]. Almost real-time data of the water depth in flooded bays provided the information needed by farmers to decide whether to open the inlets to raise the water level when low temperatures were predicted/forecast. This measure (water depth) along with information about each bays’ dimensions enabled the platform to estimate the amount of water needed to refill the water losses by water evaporation and transpiration of plants (ET_c_) to maintain the desired water depth within each bay.

### 4.1. Performance and Robustness of IRRISENS

**Figure 9:**
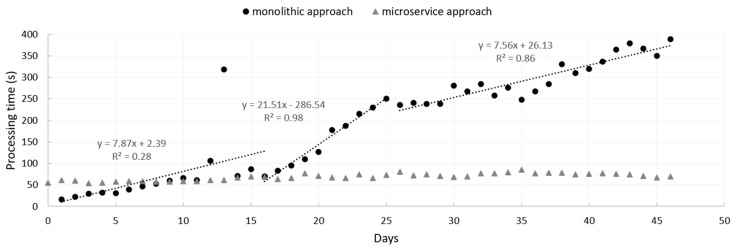
The latency of the ASS “crop parameter” microservice in a rice farm when designed to perform individually for each farm (microservice-based approach) and as a monolithic service.

The comparison between the latency of the “crop parameter” ASS that collects, processes the raw data, and cleans and updates the dataset when configured as both a microservice and as a monolithic service is illustrated in Figure 9. In both cases, IoT loggers sent the same kind of data and a similar number of parameters: 39 and 37 sensor measurements per reading cycle in the cotton and rice farms, respectively. The results show that there was a trend towards a higher processing time as the season progressed when the “crop parameter” ASS was configured as a microservice. This was an expected result because due to the cleaning process algorithm, an increase in dataset to be processed over time has an increasing effect on latency. All the microservices designed to monitor specific parameters of a farm had a similar behaviour and processing time as that illustrated for the “crop parameter” ASS in Figure 9. The increase in data flow due to the logger sending data to the platform every 20 s from day 16 to day 25 did not have a significant effect on the latency when the ASS was configured as a microservice. However, a significant increase in latency from ~70 s to 250 s was observed for the same period when the monolithic design was used instead (Figure 9). The immediate impact of this failure was that the monitoring process time in all farms increased dramatically even when the problem that caused the failure was solved. Although processing times oscillated during the season in both approaches tested (as individual microservices and as a monolithic service), in the monolithic approach, the processing time was drastically impacted by failures that occurred in any of the farms. The microservice-based approach followed in IRRISENS isolated failures that could occur in any individual farm, avoiding performance impacts on microservices in other farms being affected. As an example, at the last date of the monitoring period (end of the growing season), the processing time in the IRRISSENS microservice approach was around 75 s (measured), while in the monolithic approach the processing time at the end of the growing season based on the linear regression showed in Figure 9 (third segment of the monolithic linear regression) would be around 1329 s.

#### Daily Latency Variation for a Typical Microservice in the Cloud

**Figure 10:**
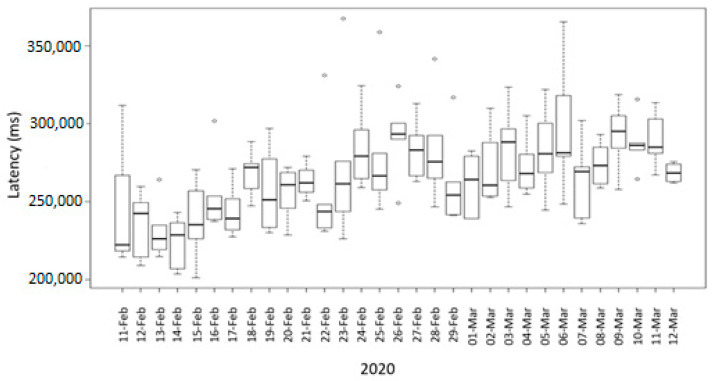
Daily latency of an ASS for one month.

Figure 10 depicts the daily latency for a month of an ASS microservice that computes and aggregates all sources of data for a crop model. During this period, the ASS microservice obtained data from the data repositories of one of the farms and executed, processed, and cleaned data every four hours to compose a new crop model. The boxplots in Figure 10 indicate the daily processing time. It is clear to see that latency variability fluctuates among days with significant variance. That was caused by the availability of resources in the cloud, particularly in the CPU at different times along the day. The evaluation of the latency of a heavy process performed by an ASS may be used to estimate the critical path regarding processing time of a complete dataflow as depicted in Figure 6.

The latency of the ASS depicted in Figure 10 when performing a heavy process was compared to the latency of an ASS when performing a simpler task such as receiving data from the field, calculating the correct sensor values, and storing data in the GCS cloud storage system (Figure 9). The latency of the ASS microservice when performing a simpler task fluctuated for a month between 50 s and 90 s, while in the ASS performing a heavy process that demands more processing time latency fluctuated between 200 s and 330 s (Figure 10). 

### 4.2. Data Heterogeneity

**Figure 11:**
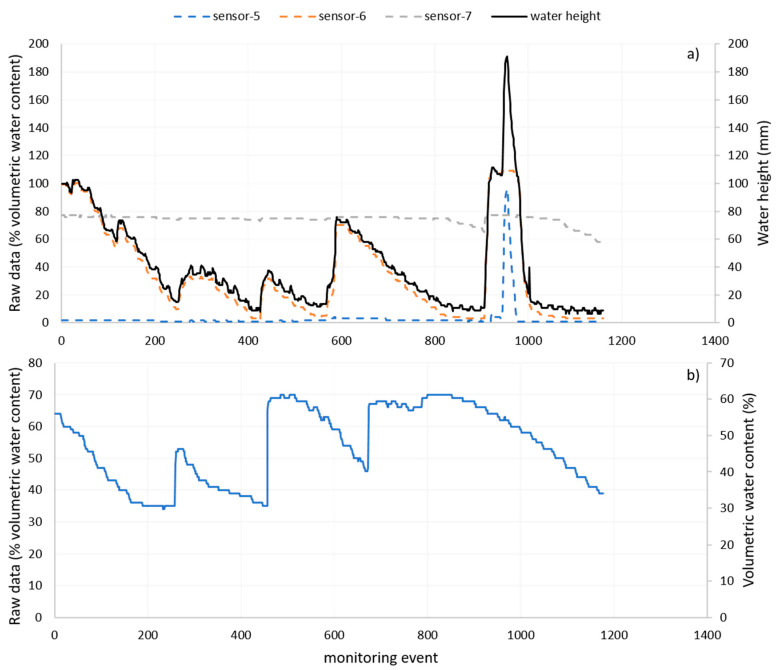
Raw data readings from the multisensor capacitance probes and processed data that is used in the crop digital model of a monitored rice (**a**) and cotton (**b**) farms during the same period.

The multisensor capacitance probe was used to show how the platform deals with data heterogeneity. Originally designed to measure volumetric soil water content the multisensor capacitance probes can be used to calculate water height in ponded crops by considering readings higher than zero from sensors installed above the soil surface. Figure 11a shows the raw data readings of a capacitance probe installed in rice and Figure 11b shows data readings of the same type of sensor installed in a cotton farm for the same period (readings for 49 days). In the case of rice, the ASS computed the three signals (raw data) to generate the water height that it is part of the crop digital model. In the cotton farm, where the capacitance sensors were used for soil moisture monitoring, the raw data were first cleaned and then stored as parameters of the crop digital model. For the period shown in Figure 11b, there were no anomalies and therefore the cleaned data was the same as the raw data.

The transmitted data in both cases have the same format and reading ranges, and the interpretation of these data may change according to the crop parameter passed into the ASS.

To evaluate the spatial and temporal heterogeneity it is necessary to consider the nature of each source of data. Remote sensing data was obtained from satellite imagery. Images were available every five days from the Sentinel-2 satellite [56] in the GMT zone. Weather data was obtained on an hourly or daily basis while soil moisture readings were always obtained every hour. To deal with such heterogeneity the microservice must interpolate and compose the remote-sensed data in the same time frame from weather service forecast and weather stations readings.

Table 3 shows the parameters obtained from the remote sensing Sentinel-2 platform and weather stations used to compose the crop digital model [50,57]. To deal with spatial heterogeneity all data is GPS referenced to fit with the monitored crop area, and data is associated with the bay identification. The spatial and temporal resolution was harmonized taking into consideration the usage of data. The remote sensing data were used to calculate the NDVI. Because Sentinel-2 imagery is available every five days, NDVI values were interpolated to fulfil the missing days. The weather data are mainly used as parameters to calculate evapotranspiration (ETo). In this case, it is important having hourly data to calculate the evapotranspiration and feed these data to the linear regression model used for forecasting. Daily data averages of relevant weather parameters are used as input in the same forecasting models to calculate the evapotranspiration one week ahead and support irrigation scheduling decisions.

## 5. Discussion

This study undertook an evaluation of an IoT platform based on fully replicable microservices used to sense and monitor soil, crop, and weather data, forecast relevant parameters, interact with external (weather and remote-sensed data providers) and third-party cloud services for planning and scheduling irrigation at commercial scales.

In terms of the requirements needed for an IoT platform applied in agriculture, the literature report only requirements for platforms applied in experimental sites or greenhouses. Several studies present requirements regarding connectivity [13,15,17,38], processing data [14,15,33], and security [34]. In this work, the connectivity already existed in the monitored crops by the structure defined in [41]. By implementing the IoT platform at four farms growing two of the most important crops in the area, rice and cotton, this study identified five requirements that IoT platforms should meet when aiming to be used in agriculture at commercial scale when a multitude of farms are to be monitored simultaneously:

1. Scalability
  - The platform should be able to sense, monitor, control, and forecast data for multiple crops across multiple farms of varied size and nature.
  - Should be able to deal with different number of monitored farms or bays within farms across growing seasons.
  - Should be allowed to scale to changes in terms of the number of external services being consumed and the number of services being offered by the platform itself.
2. Flexibility
  - It must be flexible enough to collect varied data from each field with different monitoring requirements.
  - Each crop may have a specific monitoring rate and sometimes some sensors need to collect more data at certain times so the IoT platform must be flexible to changes in monitoring rates.
  - The platform should cater for different business requirements across commercial farms.
3. Heterogeneity
  - The IoT platform must be able to receive data from different sources and locations, each one with its own local time.
  - It must be able to deal with data heterogeneity when the same type of IoT sensor is used to provide different data depending on each farm needs.
  - The platform should be able to integrate with heterogeneous communication technologies and protocols.
4. Robustness to failure
  - The platform must be robust against communication failure that may happen due to extreme weather conditions or excessive distance between sensors and data receiving points. Intermittent communication failures may happen especially in farms located in remote areas.
  - The IoT platform must be robust against unexpected failures of the IoT devices (malfunction, issues caused by animals, etc.) and must include specific procedures to monitor them.
  - Failures or changes in one farm should not compromise the applications of the platform to other farms.
5. Security
  - Data obtained from each farm should be isolated in storage and processing because of commercial privacy. Data transmission and storage should be secure from any vulnerabilities and cyber-attacks.
  - Each farmer/agronomist should be provided with a secure way to login into the platform and being able to share data when necessary with stakeholders, independent of their physical location or affiliation.
  - Farmers should be made aware of any data transparencies that might exist between the farm and the service providers.

The requirements presented in this work reflect the needs of farmers/agronomists that implemented IRRISENS for irrigation scheduling support decision-making process. The scalability and flexibility of IRRISENS meet the requirements presented in [14,15,33]. The security requirements of IRRISENS expand the discussion presented in [34] since it presents the requirements that farmers are concerned with when using an IoT platform to monitor their farms and potentially control a specific management aspect such as irrigation. One contribution of this work is in regards to the robustness of the platform to possible failures of loggers since the application of the IoT platform in commercial-scale crops makes it suitable to situations not explored in experimental sites or greenhouses.

Most IoT platforms applied in agriculture reported in the literature present cloud-centric architectures (see Table 1) and a monolithic approach as described in [11,32]. This architecture-based approach is not recommended for the context where IRRISENS was evaluated because the platform would be vulnerable to failures that could occur in individual farms. In the study here presented, we used a microservice-based approach similar to the microservice-based architectures discussed in [23,24,32], focused on message flow and the structure supporting that. A digital model was also present in the platforms introduced in [40] and in [22]. In [22], the platform was used to improve the management of crop plots and monitor crop needs for irrigation purposes and its core was the NGSI-LD broker. In the work here presented, however, IRRISENS introduces a “digital model” and “crop parameter” microservices as the core of the microservice dataflow. In [40], the platform was used for managing irrigation and differed from that proposed here in that it was structured following a monolithic approach. Further, how the platform made use of the digital model was not described in [40]. The current work not only describes the platform architecture and functioning but also evaluated the platform in a truly commercial scale scenario being used by commercial farmers. Compared to other platforms following a monolithic approach, the microservice-based architecture provided the advantage of preventing failures occurring in an individual farm, such as communication between the smart sensing devices and the cloud, malfunction of the equipment due to damage caused by adverse weather conditions or animals, common in field conditions, affecting other monitored farms. This architecture also provides an effective way to deal with data heterogeneity. Using microservices associated with cloud services, such as messages and storage mechanisms, creates a dataflow where the crop digital model is the digital representation of the physical environment under monitoring and control.

Platforms presented in other studies [11,16] have raised the importance of dealing with data heterogeneity. These studies, however, did not report how the platform should deal with other types of heterogeneity such as the spatial and temporal heterogeneity resulting from gathering data from different sources (external services). IRRISENS proposes elements to deal with all these types of heterogeneity in order to build the digital crop model. In [40], the platform makes use of several models in a monolithic platform but does not represent the entity being monitored, but rather evaluates the status of a crop, process data, and provides decision support.

The Agriculture 4.0 concept is related to the concept of Industry 4.0 as introduced in [14,32]. The IRRISENS architecture was evaluated according to the industry maturity level. The platform presents computerization and connectivity (levels 1 and 2 of the maturity level) as well as visibility (level 3), because the crop digital model could be considered as the digital shadow of the crop itself. Since the platform enables the users to evaluate the evolution of the crop during the season (NDVI, ETc, etc.) it can be considered that it also presents transparency, which is known as level 4 of the industry maturity level. The use of weather forecasts and linear regression models to evaluate crop parameters is still in a very early stage and cannot be considered as predictive capability and adaptability as presented within the Industry 4.0 concept.

Automatic irrigation control in surface-irrigated crops remains a challenge because it is not possible to control all the irrigation parameters in a deterministic manner, as done in traditional industrial control environments. Parameters important to irrigation planning, such as precipitation can only be forecast and the accuracy of these predictions are usually very low. Furthermore, the soil may be considered as a complex system [58] in which there are emergent behaviours that need to be taken into consideration for effective irrigation control.

**Figure 12:**
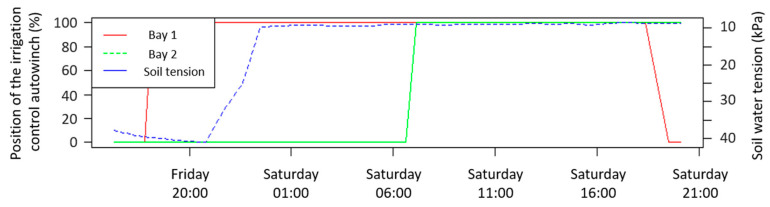
Position of the irrigation control autowinches (100% = fully open; 0% = closed) in bays 1 and 2.

The IRRISENS platform has a planning and irrigation control option that enables farmers/agronomists to monitor the soil moisture status or water heights at each bay as well as the status of the irrigation control autowinches. This means that the platform has the capability to monitor the percentage that a specific gate is open (0 to 100%) and open or close the gates to start or stop irrigation, depending on the parameters monitored (soil moisture and water height). The platform has an ASS responsible to receive the irrigation planning information from the user and send it to the external cloud to undertake effective control of the devices using a property technology. The ASS has an interface to a web service and MQTT messages and sends a set of instructions to the devices in the field. Figure 12 illustrates the position of the irrigation control autowinches during an irrigation event and the evolution of the soil water tension measured at 0.20 m depth. The position 100% represents the gate at a fully opened state. The main channel gate must be fully opened during the whole irrigation event to enable water to access the different bays. Bay 1 is the first bay to receive water. When the gate between bays 1 and 2 opens, water in bay 1 flows to the next bay, which indicates the end of bay 1 irrigation. Evaluating the irrigation times for each bay, it is possible to see that even after saturation was reached at 0.20 m depth as indicated by the sensors (soil moisture above −10 kPa), the agronomist did not open the gate until seven hours later. The main gate remained open for 24 h to irrigate the four bays.

Using IoT platforms such as IRRISENS to provide automation based on sensor feedback has the potential to ensure that optimal decisions are made that minimize irrigation water use and increase profitability. However, end users need to be confident in the system robustness. The microservices approach, such as used in IRRISENS and evaluated in this research, was found to be robust in these commercial farm environments and to offer this functionality to the end user.

## 6. Conclusions

This work presented and evaluated the IRRISENS platform designed with a microservice-based architecture for monitoring, planning, and scheduling irrigation at a commercial scale. Five main requirements for IoT platforms to be used in agriculture at commercial scale were identified from implementing the IoT platform in rice and cotton production: scalability, flexibility, heterogeneity, robustness to failure, and security. The platform was able to address all these requirements.

The microservices that compose the data flow presented affordable scalability and adaptability to monitor four farms and two different crops, each one with its own particularities. As ongoing work, the IRRISENS platform has several features to be designed to fit additional requirements, including a new set of ASS microservices to enable automatic control of irrigation gates based on crop parameters and ensure that optimal decisions are made that minimize water use and increase profitability. To automatically control irrigation of broadacre crops at commercial scales, other parameters apart from those monitored in this study should be considered. This is because this decision is not always based only on the plant water needs and other aspects, such as fertilizer application or availability of water, need to be captured as well.

The use of a standard ontology may be an important improvement to store data and correlate these with a common syntax that would be exchanged between platforms related to the same farm. However, an initial investigation indicates that the commercial platforms do not follow a common ontology, which highlights the importance to have microservices to act as the interface between the IRRISENS platform and external services. Future work for the improvement of the platform includes mapping farmer attitudes and using this knowledge in irrigation planning algorithms and also evaluating new approaches to identify the representative locations to install point source smart IoT devices in the field to represent whole irrigation field parameters.

## Figures and Tables

**Table 2:** Details of the farms and parameters monitored at each of them.

Farm	Area (ha)	Interval (min)	Weeks Monitored	Parameters Monitored
Cotton 1	2.7	60	24	Soil water tension at 0.20 cm depth Soil temperature at 0.20 cm depth Volumetric water content (%), salinity (dS m^−1^) and temperature (°C) from 0–1.2 m at 0.10 m intervals
Cotton 2	22.0	60	22	
Rice 1	33.4	60	16	Water height (mm) Soil, water, and air temperature (°C)
Rice 2	10.0	60	14	

**Table 3:** Spatial and temporal heterogeneity in remote sensing and weather forecast data.

Crop Parameter	Temporal Resolution	Spatial Resolution	Parameter	Spatial Resolution
NDVI	5 day	Bay area	Band 4	10 m
			Band 8	20 m
ETo	1 h	Bay area	Temperature, humidity, wind speed	Farm area (local weather station)
ETo forecast	1 day	Bay area	Temperature, humidity, wind speed	City area
